# Two new species of *Veronaea* (*Herpotrichiellaceae*, *Chaetothyriales*) from Guizhou Province, China

**DOI:** 10.3897/mycokeys.139.206673

**Published:** 2026-08-25

**Authors:** Wangming Zhang, Haifei Long, Xiaoyang Liu, Xiaoyu Song, Xinzhong Zhou, Yujie Zhang, Qinying Feng

**Affiliations:** 1 Beijing Jishuitan Hospital Guizhou Hospital, Guiyang 550014, China Beijing Jishuitan Hospital Guizhou Hospital Guiyang China

**Keywords:** Asexual morph, *
Eurotiomycetes
*, phylogeny, taxonomy

## Abstract

Freshwater and terrestrial ecosystems harbor a rich diversity of fungi, many of which remain poorly explored, particularly within the order *Chaetothyriales*. Four hyphomycetous isolates were obtained from freshwater and terrestrial habitats in Guizhou Province, China. Their morphological characteristics were examined, and multilocus phylogenetic analyses based on ITS, LSU, SSU, and *tub2* were performed to clarify their taxonomic positions. Phylogenetic reconstruction indicated that the isolates represent two distinct species within *Veronaea* (*Herpotrichiellaceae*, *Chaetothyriales*), which are herein described as *V. guizhouensis* and *V. terrestris*. Comprehensive descriptions, illustrations, and morphological comparisons with related taxa are provided. These findings expand the known diversity of *Veronaea* and underscore the significance of freshwater and terrestrial woody substrates as reservoirs of novel fungal taxa.

## Introduction

The family *Herpotrichiellaceae* Munk (*Eurotiomycetes*) was established by Munk (1953), with *Herpotrichiella* designated as the type genus. Currently, 26 genera are accepted within *Herpotrichiellaceae*, viz., *Aciculomyces*, *Aculeata*, *Atrokylindriopsis*, *Brycekendrickomyces*, *Capronia*, *Cladophialophora*, *Eurodistus*, *Exophiala*, *Fonsecaea*, *Marinophialophora*, *Melanoctona*, *Metulocladosporiella*, *Minimelanolocus*, *Neoherpotrichiella*, *Neosorocybe*, *Neoveronaea*, *Petriomyces*, *Phaeoannellomyces*, *Phialophora*, *Pleomelogramma*, *Rhinocladiella*, *Sorocybe*, *Thysanorea*, *Tiankengomelania*, *Valentiella*, and *Veronaea* (Wijayawardene et al. 2020; Hyde et al. 2024; Madhushan et al. 2025; Senwanna et al. 2025). The anamorph–teleomorph relationships within the family were comprehensively elucidated by Müller et al. (1987) and Untereiner et al. (1995).

The genus *Veronaea* Cif. & Montemart. (*Herpotrichiellaceae*, *Chaetothyriales*) was established by Cifferi and Montemartini (1958), with *V. botryosa* designated as the type species, which was originally isolated from a decayed palm rachis (*Arecaceae*) in Italy (Arzanlou et al. 2007). The MycoBank database records 40 taxa under *Veronaea*; however, ten species, viz. *Veronaea apiculata*, *V. bambusae*, *V. caricis*, *V. elliptica*, *V. gorakhpurensis*, *V. harunganae*, *V. indica*, *V. musae*, *V. parvispora*, and *V. verrucosa*, have been transferred to other genera. Currently, 28 species are accepted in the genus *Veronaea*; however, molecular data are available for only 14 species, viz., *V. aquatica*, *V. botryosa*, *V. brunnea*, *V. brunneicolor*, *V. compacta*, *V. endolichena*, *V. japonica*, *V. owensiae*, *V. parabrunnea*, *V. parasiticola*, *V. pinangae*, *V. polyconidia*, *V. submersa*, and *V. yosanoae* (Tan and Shivas 2025; Crous et al. 2026; Tan et al. 2026; Xiong et al. 2026). Consequently, the phylogenetic diversity and species boundaries within the genus remain incompletely resolved, and additional collections supported by morphological observations and DNA sequence data are needed to clarify its taxonomy. The asexual morph of *Veronaea* is characterized by polyblastic, terminally integrated, cylindrical, solitary, pale brown conidiogenous cells and smooth-walled, septate, cylindrical to pyriform, pale brown to brown conidia (Arzanlou et al. 2007; Vicente et al. 2008; Badali et al. 2013; Bonifaz et al. 2013; Döğen et al. 2013). Ecologically, members of the genus have been reported from diverse substrates, including terrestrial plant material, freshwater habitats, lichens, and human clinical specimens, indicating a broad ecological amplitude. Nevertheless, only the asexual morph has been documented to date, and the sexual morph remains unknown (Crous et al. 2026; Tan et al. 2026). The limited molecular sampling, together with the absence of sexual morph data, continues to hinder a comprehensive understanding of the taxonomy, evolution, and diversity of *Veronaea*.

In this study, four hyphomycetous isolates, representing two distinct taxa within *Veronaea* (*Herpotrichiellaceae*, *Chaetothyriales*), were obtained from freshwater and terrestrial substrates in Shuitian Town, Wudang District, Guizhou Province, southwestern China. Based on detailed morphological observations, illustrations, and phylogenetic analyses using maximum likelihood and Bayesian inference based on a combined dataset comprising ITS, LSU, SSU, and *tub2* sequences, two novel species are proposed: *Veronaea guizhouensis* and *V. terrestris*.

## Materials and methods

### Sample collection and specimen examination

Specimens of decaying wood were collected from freshwater and terrestrial habitats in Shuitian Town, Wudang District, Guizhou Province, China, on 22 October 2025. Samples were taken to the laboratory in plastic bags, labeled with collection details, including locality, habitat, and date (Rathnayaka et al. 2025). Samples were incubated in plastic boxes lined with moistened tissue at room temperature for 1–2 weeks (Yang et al. 2023). The samples were examined using a stereomicroscope (SMZ 800, Nikon, Japan). Micromorphological characters were captured using an EOS 90D digital camera attached to an ECLIPSE Ni compound microscope (Nikon, Japan). Measurements of conidiophores, conidiogenous cells, and conidia were carried out using the Tarosoft (R) Image Frame Work program.

### Isolation and material deposition

Single-spore isolation was performed following the method described by Senanayake et al. (2020). The germinated conidia were aseptically transferred to fresh potato dextrose agar (PDA; Oxoid, CM0139) and incubated at room temperature for 31–35 days. Morphological characteristics of the fungal mycelium on PDA, including color, shape, size, margin, and elevation, were documented. Dried fungal specimens were deposited in the Herbarium of Guizhou Academy of Agriculture Sciences (Herb. **GZAAS**), Guiyang, China. Pure cultures were deposited at the Guizhou Culture Collection (**GZCC**), Guiyang, China. Descriptions of the new taxa were uploaded to the Faces of Fungi webpage following the guidelines of Jayasiri et al. (2015). The new species were registered in the MycoBank database (https://www.mycobank.org/), and MycoBank numbers were obtained.

### DNA extraction, PCR amplification, and sequencing

The mycelium, freshly scraped from living cultures, was transferred to 1.5 mL microcentrifuge tubes and stored in a refrigerator at –20 °C. Genomic DNA was extracted using the Biospin Fungus Genomic DNA Extraction Kit (BioFlux, China), following the manufacturer’s protocol. The primer pairs ITS5/ITS4 (White et al. 1990), LR0R/LR5 (Vilgalys and Hester 1990), NS1/NS4 (White et al. 1990), and Bt2a/Bt2b (Glass and Donaldson 1995) were used to amplify the ITS, LSU, SSU, and *tub2* regions, respectively. PCR amplification was performed in a 25 μL reaction volume, consisting of 13.5 μL of 2× Taq PCR Master Mix (China; containing Taq DNA polymerase, dNTPs, MgCl_2_, and reaction buffer), 1 μL of each primer, 1 μL of template DNA, and 8.5 μL of ddH_2_O. The PCR conditions for ITS, LSU, and SSU amplification were as follows: initial denaturation at 95 °C for 5 min, followed by 35 cycles of denaturation at 94 °C for 30 s, annealing at 52 °C for 45 s, elongation at 72 °C for 1 min, and final extension at 72 °C for 10 min. The PCR conditions for *tub2* amplification were as follows: initial denaturation at 94 °C for 3 min, followed by 40 cycles of denaturation at 94 °C for 45 s, annealing at 56 °C for 50 s, elongation at 72 °C for 1 min, and final extension at 72 °C for 10 min. The PCR products were purified and sequenced by Sangon Biotech (Shanghai, China) Co., Ltd.

### Phylogenetic analyses

The forward and reverse sequences generated in this study were checked and assembled using BioEdit v. 7.0.5.3 (Hall 1999) and SeqMan v. 7.0.0 (Swindell and Plasterer 1997). Similar taxa were identified through BLASTn searches conducted on the NCBI website (https://blast.ncbi.nlm.nih.gov/Blast.cgi). Multiple sequence alignments for each locus dataset were performed using MAFFT v. 7.473 (https://mafft.cbrc.jp/alignment/server/, Katoh et al. 2019) and visually inspected in AliView (Larsson 2014). The ITS, LSU, SSU, and *tub2* alignments were trimmed using trimAl v. 1.2rev59 (Capella-Gutiérrez et al. 2009) and subsequently merged using SequenceMatrix v. 1.7.8 (Vaidya et al. 2011) (Table 1).

**Table 1.** Taxa used in this study, along with their corresponding GenBank accession numbers.

**Taxon**	**Strain/specimens**	**GenBank accession numbers**				**References**
		**ITS**	**LSU**	**SSU**	***tub*2**	
*Cyphellophora laciniata*	CBS 190.61^T^	EU035416	FJ358239	FJ358307	JQ766329	Crous et al. (2007)
*Cyphellophora suttonii*	CBS 449.91^T^	KC455243	KC455256	KC455300	KC455226	Réblová et al. (2013)
*Exophiala bonariae*	CBS 139957^T^	JX681046	KR781083	NA	NA	Isola et al. (2013)
*Exophiala candelabrata*	FMR 18336^T^	ON009851	ON009931	NA	ON491591	Torres-Garcia et al. (2023)
*Exophiala opportunisticica*	CBS 109811^T^	KF928437	KF928501	NA	JN112486	Attili-Angelis et al. (2014)
*Exophiala pisciphila*	CBS 537.73^T^	DQ826739	AF050272	DQ823108	JN112493	James et al. (2006)
*Exophiala radicis*	P2854^T^	KT099204	KT723448	KT723453	KT723463	Maciá-Vicente et al. (2016)
*Exophiala salmonis*	CBS 157.67^T^	JF747137	AY213702	EF413608	JN112499	de Hoog et al. (2014)
*Fonsecaea monophora*	CBS 102243	EU938579	FJ358247	FJ225747	EU938542	Najafzadeh et al. (2009)
*Thysanorea aquaticus*	MFLUCC 15-0414	KR215607	KR215612	KR215617	NA	Liu et al. (2015)
*Thysanorea clavatus*	DLU 3022^T^	MT271774	MT271772	MT271777	NA	Wan et al. (2021)
*Thysanorea papuana*	CBS 212.96^T^	MH862572	MH874198	NA	NA	Vu et al. (2019)
*Thysanorea submersus*	KUMCC 15-0206^T^	KX789212	KX789215	NA	NA	Hyde et al. (2016)
*Thysanorea thailandensis*	MFLUCC 15-0971^T^	MG922573	MG922577	MG922581	NA	Dong et al. (2018)
*Veronaea aquatica*	JAUCC2549^T^	MW046892	MW046893	NA	MW248394	Chandrasiri et al. (2021)
*Veronaea botryosa*	CBS 254.57^T^	MH857711	MH869255	JN856021	JN112505	Vu et al. (2019)
*Veronaea botryosa*	MFLUCC 11-0072	MG922570	MG922574	MG922578	NA	Dong et al. (2018)
*Veronaea botryosa*	CBS 102593	KF928429	KF928493	NA	KF928557	Attili-Angelis et al. (2014)
*Veronaea botryosa*	CBS 572.90	MH862237	MH873920	NA	NA	Vu et al. (2019)
*Veronaea botryosa*	GZCC 19-0557	OP377853	OP377938	OP378020	NA	Yang et al. (2023)
*Veronaea brunnea*	CBS 587.66	MH858890	KX712342	JN856013	JN112442	Crous et al. (2026)
*Veronaea brunneicolor*	CGMCC 3.23628^T^	OQ645271	OQ645285	OQ645278	OQ696284	Chang et al. (2025)
*Veronaea compacta*	CBS 268.75^T^	EU041819	EU041876	NA	NA	Arzanlou et al. (2007)
*Veronaea endolichena*	SDBR-CMU507^T^	PQ525393	PQ525401	PQ525409	PQ523735	Senwanna et al. (2025)
*Veronaea endolichena*	SDBR-CMU508	PQ525394	PQ525402	PQ525410	PQ523736	Senwanna et al. (2025)
*Veronaea endolichena*	SDBR-CMU509	PQ525395	PQ525403	PQ525411	PQ523737	Senwanna et al. (2025)
***Veronaea guizhouensis***	**GZCC 25-27598^T^**	**PZ296703**	**PZ296711**	**PZ296762**	**PZ688209**	**This study**
***Veronaea guizhouensis***	**GZCC 25-27599**	**PZ296704**	**PZ296712**	**PZ296763**	**PZ684738**	**This study**
*Veronaea japonica*	CBS 776.83^T^	EU041818	EU041875	NA	NA	Arzanlou et al. (2007)
*Veronaea owensiae*	BRIP 76889a^T^	PX909694	PX909686	NA	NA	Tan et al. (2026)
*Veronaea parabrunnea*	CBS 153520	PZ221329	PZ221373	NA	PZ228740	Crous et al. (2026)
*Veronaea parasiticola*	CBS 153523	PZ221330	PZ221374	NA	PZ228741	Crous et al. (2026)
*Veronaea pinangae*	MFLUCC 25-0212^T^	PV578268	PV578436	NA	PV607970	Xiong et al. (2026)
*Veronaea polyconidia*	CGMCC 3.25589^T^	OR807862	OR807868	OR807865	OR817660	Su et al. (2023)
*Veronaea polyconidia*	UESTCC 23.0138	OR807863	OR807869	OR807866	OR817661	Su et al. (2023)
*Veronaea polyconidia*	UESTCC 23.0139	OR807861	OR807867	OR807864	OR817659	Su et al. (2023)
*Veronaea submersa*	GZCC 25-0693^T^	PV982848	PV982828	PV982863	NA	Bao et al. (2025)
***Veronaea terrestris***	**GZCC 25-27600**	**PZ296705**	**PZ296713**	**PZ296764**	**PZ688207**	**This study**
***Veronaea terrestris***	**GZCC 25-27601^T^**	**PZ296706**	**PZ296714**	**PZ296765**	**PZ688208**	**This study**
*Veronaea yosanoae*	BRIP 75683a^T^	PX127649	PX127653	NA	NA	Tan et al. (2026)

Note: “^T^” indicates ex-type strains. Newly generated sequences are shown in bold. “NA” indicates data unavailable in GenBank.

Maximum likelihood (ML) phylogenetic analyses were performed using IQ-TREE v. 3.1.3 for Windows (Zeng et al. 2023). Bayesian inference (BI) was performed using the One-click Fungal Phylogenetic Tool (OFPT) method described by Zeng et al. (2023). The aligned FASTA file was converted to NEXUS format using AliView (Daniel et al. 2010). The best-fit evolutionary model for each dataset was determined using MrModeltest v. 2.3.10 (Swofford 2002; Nylander 2004). The SYM+I+G substitution model was selected for ITS, the GTR+I+G model for LSU, the HKY+I model for SSU, and the GTR+G model for *tub2*. The posterior probabilities (PP) were determined based on Bayesian Markov chain Monte Carlo (BMCMC) sampling (Huelsenbeck and Ronquist 2001). Four simultaneous Markov chains were run for 10,000,000 generations, and trees were sampled every 1,000^th^ generation. The burn-in phase was set at 25%, and the remaining trees were used to calculate posterior probabilities. Phylogenetic trees were visualized using FigTree v. 1.4.4 and further edited in PowerPoint. The photoplate was prepared using Adobe Photoshop CS6 software (Adobe Systems, USA).

### Phylogenetic analysis results

**Figure 1.**
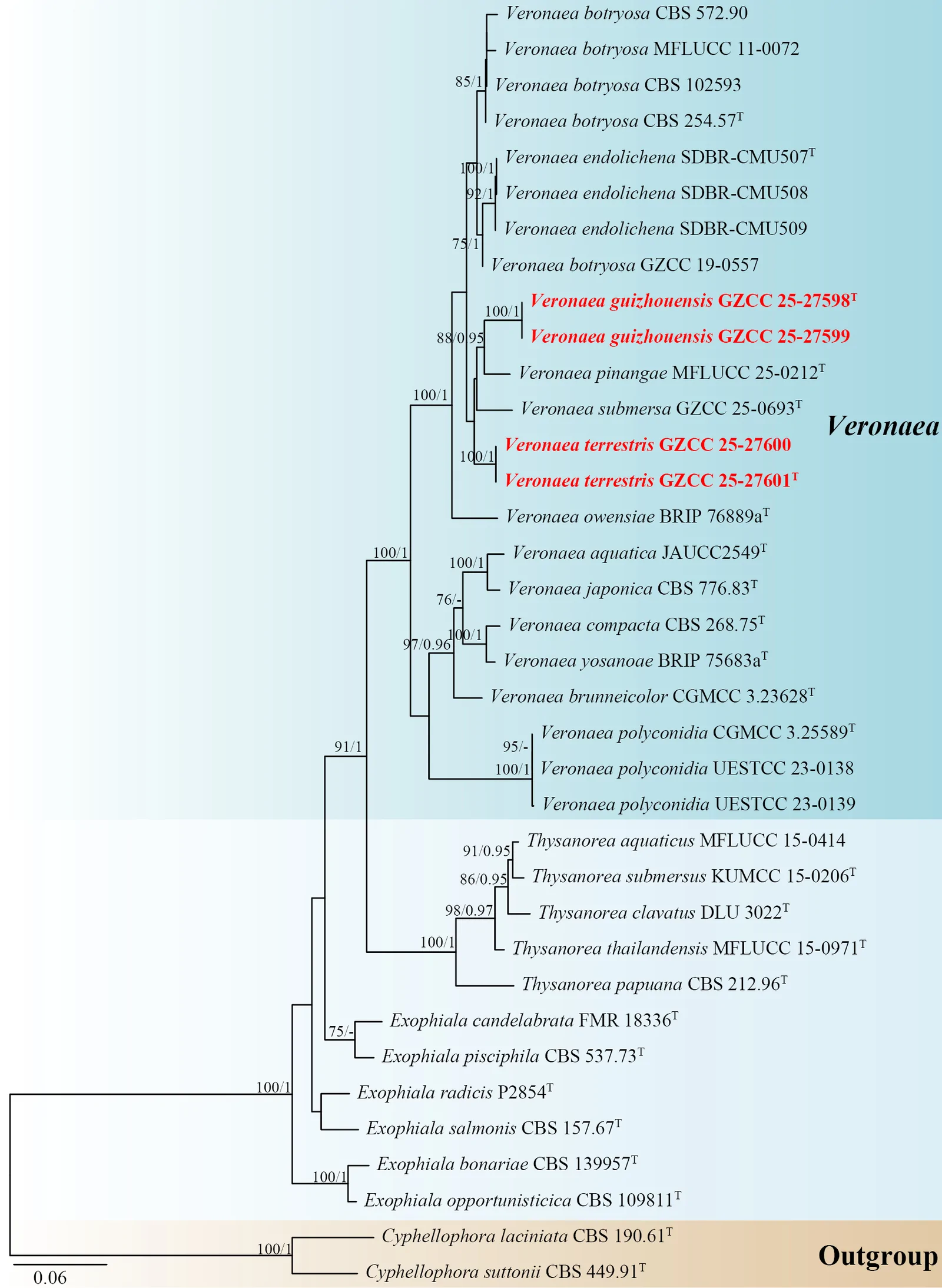
Phylogenetic tree inferred from maximum likelihood (ML) analysis based on the combined ITS, LSU, SSU, and *tub2* sequence dataset. Bootstrap support values ≥ 75% for ML (left) and Bayesian posterior probabilities (PP) ≥ 0.95 (right) are indicated at the nodes. A hyphen (-) indicates a posterior probability lower than 0.95 for BI. The tree is rooted with *Cyphellophora laciniata* (CBS 190.61) and *C. suttonii* (CBS 449.91). Ex-type strains are indicated by “^T,^” and newly obtained isolates are highlighted in bold red.

The phylogenetic positions of the four novel strains were evaluated using a multilocus phylogenetic approach. The concatenated alignment comprised 3,047 characters, including ITS (positions 1–654), LSU (655–1,548), SSU (1,549–2,569), and *tub2* (2,570–3,047), representing a total of 36 taxa. Both the ML and BI analyses of the concatenated ITS, LSU, SSU, and *tub2* datasets yielded similar tree topologies. Fig. 1 illustrates the best-scoring ML tree, with a final likelihood value of −11,467.306. Species recognition and the justification for establishing new species followed the guidelines proposed by Jeewon and Hyde (2016) and Chethana et al. (2021).

Based on the multilocus phylogenetic tree (Fig. 1), the collections were resolved as two distinct *Veronaea* species within the family *Herpotrichiellaceae (Chaetothyriales)*. Isolates GZCC 25-27598 and GZCC 25-27599 formed a sister clade to *Veronaea pinangae* (MFLUCC 25-0212) with 88% ML/0.95 PP support. Furthermore, isolates GZCC 25-27600 and GZCC 25-27601 clustered with *V. guizhouensis* (GZCC 25-27598 and GZCC 25-27599), *V. pinangae* (MFLUCC 25-0212), and *V. submersa* (GZCC 25-0693).

## Taxonomy

### Veronaea guizhouensis

Taxon classificationFungiChaetothyrialesHerpotrichiellaceae

W. M. Zhang & Q. Y. Feng sp. nov.

092D21EC-4A83-5D2E-BD0F-B7E3EC00E39B

905858

Fig. 2

#### Etymology.

"*guizhouensis*" refers to the place “Guizhou Province” from where the holotype was collected.

#### Holotype.

GZAAS 25-0789.

#### Description.

***Saprobic*** on decaying wood in a freshwater habitat. **Sexual morph** Undetermined. **Asexual morph** Hyphomycetous. ***Colonies*** on natural substrate superficial, effuse, gregarious, hairy, brown. ***Mycelium*** immersed in the substratum, consisting of subhyaline to pale brown, smooth hyphae. ***Conidiophores*** 177–209 × 5–6.5 μm (x̄ = 189 × 6 μm, *n* = 20), mononematous, macronematous, branched, erect, straight or flexuous, cylindrical, smooth-walled, pale brown to brown, pale brown to subhyaline at the apex, septate, slightly constricted at septa, thin-walled. ***Conidiogenous cells*** 39–63.5 × 3–5 μm (x̄ = 54 × 3.5 μm, *n* = 25), terminal, polyblastic, sympodial, cylindrical, subhyaline to pale brown, rachis with crowded, flat to slightly prominent denticles. ***Conidia*** 7–14 × 2.5–5 μm (x̄ = 10 × 3.5 μm, *n* = 25), acropleurogenous, cylindrical to pyriform, rounded at apex and truncate at base, straight, 1–3-septate, constricted at the septa, subhyaline to olivaceous-brown, solitary, smooth, thin-walled. ***Conidial*** seccession schizolytic.

**Figure 2.**
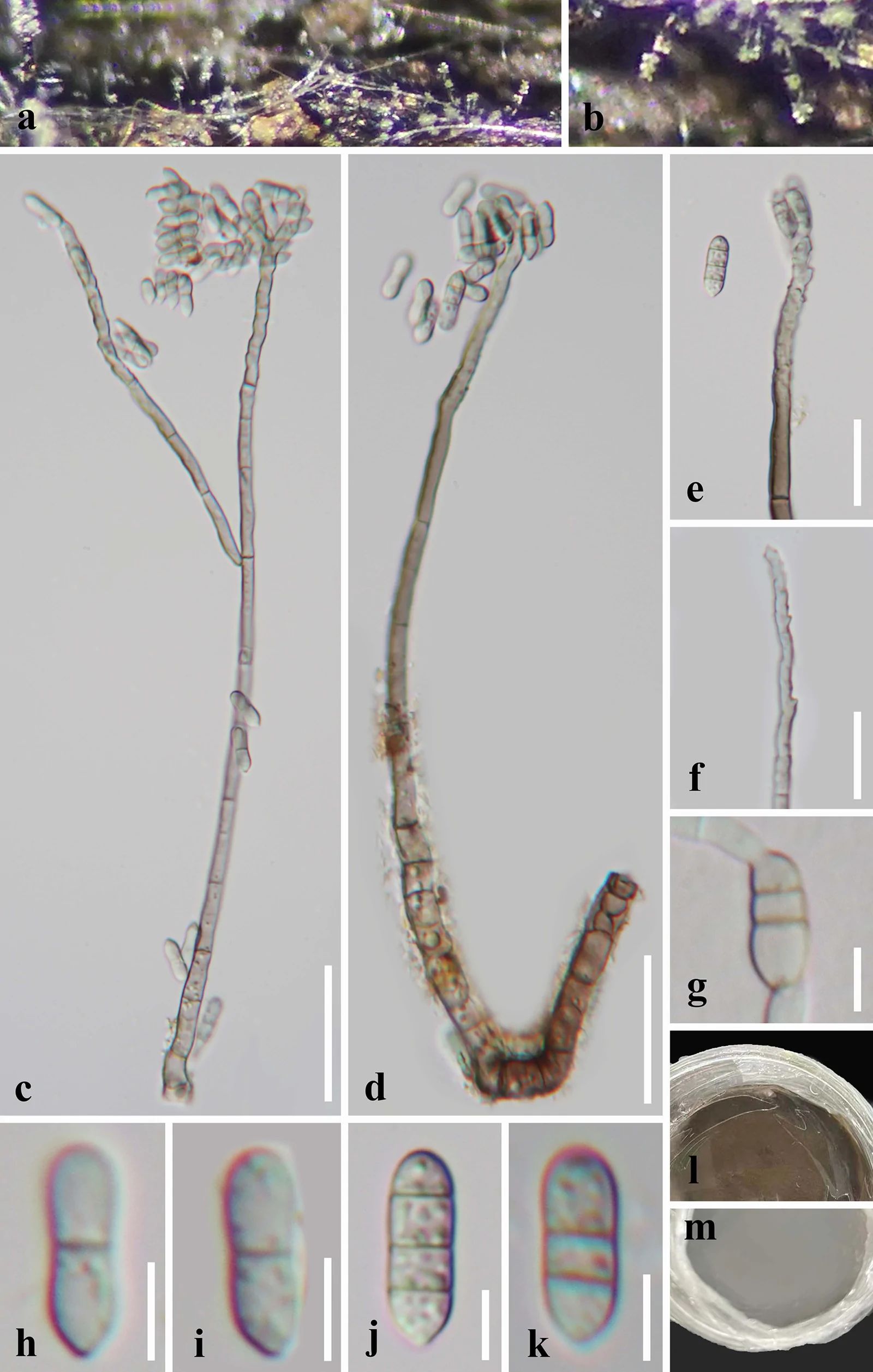
*Veronaea guizhouensis* (GZAAS 25-0789, holotype). **a, b**. Colonies on the host surface; **c, d**. Conidiophores, conidiogenous cells, and conidia; **e, f**. Conidiogenous cells and conidia; **g**. Germinated conidium; **h–k**. Conidia; **l, m**. Colonies on PDA from above and below. Scale bars: 30 μm (**c, d**); 20 μm (**e, f**); 5 μm (**g–k**).

#### Culture characteristics.

Conidia germinated on PDA within 9 h, producing germ tubes. Colonies on PDA reached 39 mm in diameter after 31 days of incubation at 25 °C, circular, with a flat surface, and an entire margin, surface brown to yellowish brown, reverse dark brown to black.

#### Material examined.

China • Guizhou Province, Guiyang City, Wudang District, Shuitian Town, on decaying wood in a freshwater habitat, 22 October 2025, Wang-Ming Zhang & Qinying Feng, GG5 (GZAAS 25-0789, holotype), ex-type living culture GZCC 25-27598; • ibid., GG9 (GZAAS 25-0790, isotype), living culture GZCC 25-27599.

#### Notes.

In the phylogenetic analyses (Fig. 1), the isolates (GZCC 25-27598 and GZCC 25-27599) formed a sister clade to *Veronaea pinangae* (MFLUCC 25-0212) with 88% ML/0.95 PP support. Pairwise nucleotide comparisons of the ITS, LSU, and *tub2* regions between *Veronaea guizhouensis* (GZCC 25-27598) and *V. pinangae* (MFLUCC 25-0212) revealed differences of 13/407 bp (3.2%, including one gap), 6/904 bp (0.7%, including two gaps), and 43/375 bp (11.5%, including eight gaps), respectively. Morphologically, *Veronaea guizhouensis* (GZAAS 25-0789) is readily distinguished from *V. pinangae* (MFLU 25-0151) by its longer conidiophores (177–209 μm vs. 160–180 μm), longer conidiogenous cells (39–63.5 μm vs. 20–23 μm), and shorter conidia (7–14 μm vs. 18–20 μm) (Xiong et al. 2026). Therefore, based on both molecular phylogenetic evidence and morphological distinctions, *Veronaea guizhouensis* is herein introduced as a novel species.

### Veronaea terrestris

Taxon classificationFungiChaetothyrialesHerpotrichiellaceae

W. M. Zhang & Q. Y. Feng sp. nov.

B1EFBFB3-4D13-5887-A833-4318F022D7C8

905859

Fig. 3

#### Etymology.

“*terrestris*’’ refers to terrestrial habitat of this fungus.

#### Holotype.

GZAAS 25-0791.

#### Description.

***Saprobic*** on decaying wood in a terrestrial habitat. **Sexual morph** Undetermined. **Asexual morph** Hyphomycetous. ***Colonies*** on natural substrate superficial, effuse, gregarious, hairy, brown. ***Mycelium*** immersed in the substratum, consisting of subhyaline to pale brown, smooth hyphae. ***Conidiophores*** 77–134 × 2.5–6 μm (x̄ = 111.5 × 4.5 μm, *n* = 20), mononematous, macronematous, unbranched, erect, straight or flexuous, cylindrical, smooth-walled, pale brown to brown, pale brown to subhyaline at the apex, septate, slightly constricted at septa, thin-walled. ***Conidiogenous cells*** 16–35 × 2.5–4 μm (x̄ = 27.5 × 3.3 μm, *n* = 30), terminal, polyblastic, sympodial, cylindrical, subhyaline to pale brown, rachis with crowded, flat to slightly prominent denticles. ***Conidia*** 9–12.5 × 3–5 μm (x̄ = 10.5 × 4 μm, *n* = 35), acropleurogenous, cylindrical to pyriform, rounded at apex and truncate at base, straight, 1–3-septate, constricted at the septa, subhyaline to olivaceous-brown, solitary, smooth, thin-walled. ***Conidial*** seccession schizolytic.

**Figure 3.**
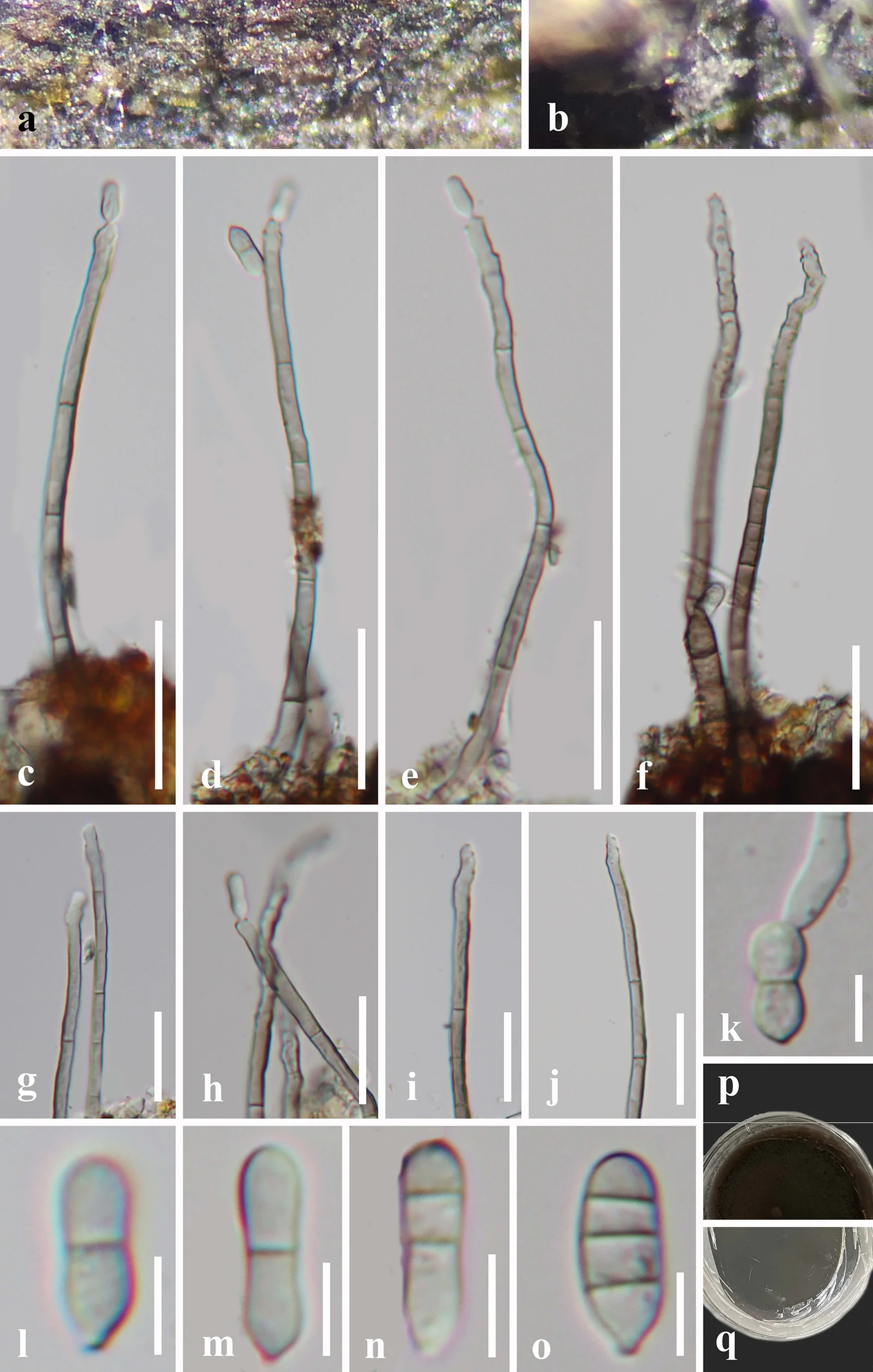
*Veronaea terrestris* (GZAAS 25-0791, holotype). **a, b**. Colonies on the host surface; **c–e**. Conidiophores, conidiogenous cells, and conidia; **f**. Conidiophores and conidiogenous cells; **g–j**. Conidiogenous cells; **k**. Germinated conidium; **l–o**. Conidia; **p, q**. Colonies on PDA from above and below. Scale bars: 30 μm (**c, d, f**); 20 μm (**e, g–j**); 5 μm (**k–o**).

#### Culture characteristics.

Conidia germinated on PDA within 11 h, producing germ tubes. Colonies on PDA reached 40 mm in diameter after 35 days of incubation at 25 °C, circular, with a flat surface, and an entire margin, surface brown to yellowish brown, reverse dark brown to black.

#### Material examined.

China • Guizhou Province, Guiyang City, Wudang District, Shuitian Town, on decaying wood in a terrestrial habitat, 22 October 2025, Wang-Ming Zhang & Qinying Feng, Q27 (GZAAS 25-0791, holotype), ex-type living culture GZCC 27-27601; • ibid., Q12 (GZAAS 25-0792, isotype), living culture GZCC 27-27600.

#### Notes.

Morphologically, *Veronaea terrestris* (GZAAS 25-0791) closely resembles *V. guizhouensis* (GZAAS 25-0789) in having mononematous, macronematous, erect, cylindrical, septate conidiophores; terminal, polyblastic, sympodial conidiogenous cells; and acropleurogenous, cylindrical to pyriform, straight, 1–3-septate conidia. However, *Veronaea guizhouensis* (GZAAS 25-0789) differs from *V. terrestris* (GZAAS 25-0791) by longer conidiophores (177–209 μm vs. 77–134 μm) and longer conidiogenous cells (39–63.5 μm vs. 16–35 μm). In the present phylogenetic analysis, *Veronaea terrestris* (GZCC 25-27600 and GZCC 25-27601) clustered with *V. guizhouensis* (GZCC 25-27598 and GZCC 25-27599), *V. pinangae* (MFLUCC 25-0212), and *V. submersa* (GZCC 25-0693), while forming a distinct lineage, indicating that these taxa represent separate species. Based on the molecular sequence comparison, the isolate (GZCC 25-27600, ex-type) differs from *V. guizhouensis* (GZCC 25-27598, ex-type) by 17/409 bp for ITS (4.2%, gaps 5 bp), 11/894 bp (1.2%, without gaps) for LSU, 4/1018 bp (0.4%, without gaps) for SSU, and 56/484 bp (11.6%, gaps 9 bp) for *tub2*. Therefore, GZCC 25-27600 and GZCC 25-27601 are identified as a new species, *Veronaea terrestris*.

## Discussion

The genus *Veronaea* currently comprises 30 species, including the two novel species described in the present study (Chang et al. 2025; Tan and Shivas 2025; Tan et al. 2026). Species of *Veronaea* have a broad geographic distribution, occurring in Australia, Brazil, China, Egypt, India, New Zealand, North America, South Africa, Thailand, and the UK (Dingley 1972; Papendrof 1976; Morgan-Jones 1982; Moustafa and Abdul-Wahid 1990; Kharwar and Singh 2004; Soares and Barreto 2008; Pan et al. 2009, 2012; Pan and Zhang 2010; Yang et al. 2023; Xiong et al. 2026). These fungi inhabit submerged wood in freshwater, soil, and various terrestrial hosts (Kharwar and Singh 2004; Arzanlou et al. 2007; Dong et al. 2018; Su et al. 2023; Bao et al. 2025; Chang et al. 2025; Tan and Shivas 2025; Crous et al. 2026; Tan et al. 2026). Among freshwater habitats, *Veronaea aquatica*, *V. botryosa*, *V. coprophila*, *V. oblongispora*, and *V. submersa* have been recorded exclusively from submerged wood (Hyde and Goh 1998; Tsui 1999; Kondo et al. 2007; Sang et al. 2011; Dong et al. 2018; Chandrasiri et al. 2021; Yang et al. 2023; Bao et al. 2025). The present study contributes to a more comprehensive understanding of *Veronaea* by documenting two previously unknown species from freshwater and terrestrial habitats. Although the genus has been reported from a wide range of geographic regions and ecological niches, freshwater representatives remain comparatively scarce. The discovery of these taxa suggests that submerged woody substrates continue to harbor overlooked fungal diversity and that the species richness of *Veronaea* in aquatic ecosystems is likely underestimated. By integrating detailed morphological comparisons with multilocus phylogenetic analyses, this study also provides additional taxonomic evidence that facilitates accurate species delimitation and improves the understanding of evolutionary relationships within the genus. Collectively, these findings enrich current knowledge of the taxonomy, phylogeny, and ecological diversity of *Veronaea* and provide a basis for future investigations of freshwater fungi.

*Veronaea* species have received increasing attention because of their potential value in pharmaceutical and agricultural biotechnology (Zhou et al. 2015; Chang et al. 2025). Previous studies have demonstrated that members of the genus are capable of producing biologically active secondary metabolites with cytotoxic properties. For example, *Veronaea brunneicolor* exhibited moderate cytotoxic activity against the human lung cancer cell lines H460 and A549 and showed inhibitory activity against several plant pathogenic fungi, highlighting its potential as a source of both anticancer compounds and antifungal agents (Chang et al. 2025). Similarly, Zhou et al. (2015) reported cytotoxic metabolites from *Veronaea*, further supporting the biotechnological potential of the genus. Despite these promising findings, the metabolic diversity and ecological roles of most *Veronaea* species remain largely unexplored. Therefore, the discovery and characterization of additional species not only improve the understanding of the taxonomy and diversity of the genus but also expand the pool of fungal resources available for future studies on natural products and other biotechnological applications.

A key to the species accepted in *Veronaea* is provided.

### Key to species of *Veronaea*

**Table:** 

1	Conidiogenous cells phialidic, with a collarette	**2**
–	Conidiogenous cells holoblastic or sympodial, without a collarette	**5**
2	Conidia aseptate	***V. parasiticola***
–	Conidia 0–1-septate	**3**
3	Conidiophores up to 350 μm long; phialides variable, flask-shaped to oblong; colonies mouse-grey	***V. brunnea***
–	Conidiophores shorter than 50 μm	**4**
4	Conidia subcylindrical to ellipsoid or subclavate, (5–)6–7(–10) × (2.5–)3 μm; conidiophores up to 50 μm	***V. parabrunnea***
5	Conidia predominantly aseptate	**6**
–	Conidia usually septate	**9**
6	Conidia oblong, 7–8 × 4–5 μm, rather thick-walled	***V. oblongispora***
–	Conidia ellipsoid, obovoid or broadly obovoid	**7**
7	Conidia broadly obovoid, 7.5–9.5 × 3–5 μm	***V. latispora***
–	Conidia ellipsoid, smaller than above	**8**
8	Conidiophores up to 350 μm long	***V. gobica***
–	Conidiophores rarely exceeding 50 μm	***V. compacta***
9	Conidia mostly 3–5-septate	**10**
–	Conidia 0–3-septate, usually 1-septate	**13**
10	Conidia 18–20 × 5–7 μm, ellipsoid to fusiform, tapering to a pointed apex	***V. pinangae***
–	Conidia smaller	**1**
11	Conidia 21.5–32.5 × 2–5 μm, cylindrical to slightly obclavate, 1–12-septate	***V. smilacis***
–	Conidia shorter than 20 μm	**12**
12	Conidiophores extremely long (1125–1515 μm); conidia mostly 3-septate	***V. polyconidia***
–	Conidiophores up to 200 μm; conidia usually 1-septate	***V. tectonae***
13	Conidia verruculose or echinulate	**14**
–	Conidia smooth	**17**
14	Conidia finely echinulate; conidiophores 400–800 μm long	***V. filicina***
–	Conidia verruculose	**15**
15	Plant pathogenic species	**16**
–	Saprobic species	***V. thylacospermi***
16	Conidia obovoidal, 4.5–18.5 μm long	***V. ficina***
–	Conidia cylindrical to obovate or ellipsoid; Conidiophores branched; conidia 7.3–19.5 × 2.6–7.5 μm	***V. grewiicola***
	Conidiophores usually unbranched; conidia 4–18.5 × 1.5–3.5 μm	***V. hippocratiae***
17	Conidiophores branched	**18**
–	Conidiophores unbranched or only rarely branched	**24**
18	Conidiophores exceeding 170 μm	**19**
–	Conidiophores less than 170 μm	**21**
19	Conidiophores 177–209 μm long; freshwater species	***V. guizhouensis***
–	Conidiophores up to 280 μm long	**20**
20	Conidia 6–11(–12) × 2.5–3.5(–4) μm; mostly 1-septate	***V. aquatica***
–	Conidia 8–14 × 2.5–4 μm; prominently constricted at septa	***V. constricta***
21	Conidiophores rough-walled	***V. endolichena***
–	Conidiophores smooth	**22**
22	Conidiogenous cells geniculate	***V. brunneicolor***
–	Conidiogenous cells not geniculate	**23**
23	Conidiophores up to 130 μm; conidia 9–17 × 2.5–4 μm	***V. carlinae***
–	Conidiophores 35–110 μm; conidia clavate, 1-septate	***V. queenslandica***
24	Conidiogenous loci with prominent geniculate scars	***V. simplex***
–	Conidiogenous loci denticulate or inconspicuous	**25**
25	Conidiophores 77–134 μm long	***V. terrestris***
–	Conidiophores usually shorter or longer than above	**26**
26	Conidiophores up to 65 μm long	***V. japonica***
–	Conidiophores 19.5–86.5 μm long, leaf pathogen	***V. hedychii***
–	Conidiophores 78–180 μm long; conidia clavate to obovoid	***V. botryosa***

## Supplementary Material

XML Treatment for Veronaea guizhouensis

XML Treatment for Veronaea terrestris

## Additional information

### Artificial Intelligence (AI) use

The authors accept full responsibility for the content of the manuscript, including the disclosure of any use of AI.

No AI tools were used in the preparation of this manuscript.

### Data availability

All of the data that support the findings of this study are available in the main text.
